# Kinetic analysis of synaptonemal complex dynamics during meiosis of yeast *Saccharomyces cerevisiae* reveals biphasic growth and abortive disassembly

**DOI:** 10.3389/fcell.2023.1098468

**Published:** 2023-02-06

**Authors:** Michael G. Pollard, Beth Rockmill, Ashwini Oke, Carol M. Anderson, Jennifer C. Fung

**Affiliations:** Department of Obstetrics, Gynecology and Reproductive Sciences, Center of Reproductive Sciences, University of California, San Francisco, San Francisco, CA, United States

**Keywords:** synaptonemal complex, meiosis, Zip1, Zip3, yeast, synapsis, *in vivo* microscopy, polymer dynamics

## Abstract

The synaptonemal complex (SC) is a dynamic structure formed between chromosomes during meiosis which stabilizes and supports many essential meiotic processes such as pairing and recombination. In budding yeast, Zip1 is a functionally conserved element of the SC that is important for synapsis. Here, we directly measure the kinetics of Zip1-GFP assembly and disassembly in live cells of the yeast *S. cerevisiae*. The imaging of SC assembly in yeast is challenging due to the large number of chromosomes packed into a small nucleus. We employ a *zip3*Δ mutant in which only a few chromosomes undergo synapsis at any given time, initiating from a single site on each chromosome, thus allowing the assembly and disassembly kinetics of single SCs to be accurately monitored in living cells. SC assembly occurs with both monophasic and biphasic kinetics, in contrast to the strictly monophasic assembly seen in *C. elegans*. In wild-type cells, once maximal synapsis is achieved, programmed final disassembly rapidly follows, as Zip1 protein is actively degraded. In *zip3Δ*, this period is extended and final disassembly is prolonged. Besides final disassembly, we found novel disassembly events involving mostly short SCs that disappeared in advance of programmed final disassembly, which we termed “abortive disassembly.” Abortive disassembly is distinct from final disassembly in that it occurs when Zip1 protein levels are still high, and exhibits a much slower rate of disassembly, suggesting a different mechanism for removal in the two types of disassembly. We speculate that abortive disassembly events represent defective or stalled SCs, possibly representing SC formation between non-homologs, that is then targeted for dissolution. These results reveal novel aspects of SC assembly and disassembly, potentially providing evidence of additional regulatory pathways controlling not just the assembly, but also the disassembly, of this complex cellular structure.

## Introduction

Meiosis is a crucial part of gametogenesis in sexually reproducing organisms. The meiotic program is unique in that replicated chromosomes find and align lengthwise along their homologous partners, exchange genetic material, and then segregate twice, resulting in haploid gametes. The pairwise alignment of homologous chromosomes ensures that genetic exchange will occur between homologs. Crossovers, or reciprocal genetic exchanges, result in physical connections between the chromosome pairs that serve to align them for proper segregation. The synaptonemal complex (SC) is the protein matrix that forms along the lengths of homologs and is thought to stabilize the paired homologs and regulate the number of recombination events that occur along the length of chromosomes (Lake and Hawley, 2021). The SC is composed of lateral elements, formed along each replicated homolog, and a central region of ordered proteins that unite these axes. The central region of the SC, but not the axes, appears to have fluid-like properties in both yeast and worms (Rog et al., 2017), where the weakly-bonded proteins can move around within the structure. The assembly of the SC is a dynamic process that appears to be aided by the pulling of chromosome ends from outside the nucleus using a connection of the chromosome to the nuclear envelope *via* the LINC complex and either microtubules or actin fibers to pull them (Alleva and Smolikove, 2017). Disassembly of the SC is coordinated with the resolution of connections between the chromosomes, and its timing is subject to cell cycle regulation (Hochwagen and Amon, 2006; Jordan et al., 2009). Since failures in meiosis can lead to infertility, miscarriages and potentially developmental problems in offspring, it is important to gain a better understanding of SC assembly and disassembly. Moreover, formation of the SC is a massive feat of molecular self-assembly, whose mechanism may hold lessons for other large-scale assembly processes in the cell.

There are three identified central region proteins in yeast. Zip1, a major component of the SC central region, is a structurally-conserved protein that was first identified in yeast (Sym et al., 1993). Zip1 and functionally analogous proteins in mice, worms, plants and mammals consist of a long coiled-coil filament with unstructured domains at either end (Page and Hawley, 2004). These transverse filaments, through the interactions of their central coiled-coil region are thought to form N-terminal tetrameric building blocks that self-assemble into the SC with the C-terminal regions interacting with the lateral elements (Dong and Roeder, 2000; Dunce et al., 2018). This configuration and the length of the coiled coil are responsible for the conserved 100 nm width of the SC (Sym and Roeder, 1995). GFP-tags inserted in the middle of Zip1 and its homologs (White et al., 2004) have been widely used to visualize chromosome dynamics during meiosis, including rapid telomere-led movements (Koszul et al., 2008) and SC fluidity (Rog et al., 2017). The other two identified central region proteins are Ecm11 and Gmc2 which facilitate the assembly of Zip1 (Humphryes et al., 2013). Gcm2 promotes the sumoylation of Ecm11 by the E3 SUMO ligases, Siz1 and Siz2 (Leung et al., 2015). The Zip1, at the N-terminus, activates the further sumoylation of Ecm11 and this positive feedback loop forms the SC (Leung et al., 2015). Voelkel-Meiman et al. (2012) using additional copies of *ZIP1* demonstrated a positive correlation between the concentration of Zip1 and speed of synapsis onset.

The initiation of SC formation appears to occur at either of two locations: 1) presumptive crossover sites and/or 2) specific chromosome domains. In budding yeast, sites of genetic exchange accumulate proteins that attract components important for SC initiation (Chua and Roeder, 1998; Agarwal and Roeder, 2000; Tsubouchi et al., 2006; Pyatnitskaya et al., 2022). A subset of these sites is likely to be responsible for most SC initiations. Yeast centromeres are also sites of SC initiation in which centromeres appear to be among the first regions to accumulate SC proteins and to initiate SC formation (Tsubouchi et al., 2008). SC initiation at centromeres is licensed only after recombination has initiated (Macqueen and Roeder, 2009). In organisms that do not rely on recombination to engage homologous chromosomes, special chromosomal sites are used to pair and initiate synapsis. In the nematode *C. elegans*, the pairing centers are present on one end of each chromosome and are responsible for assembling SC along the homologs. In this case, SC formation is independent of recombination (Dernburg et al., 1998). In the fly, *D. melanogaster*, SC initiation occurs at centromeres and is also independent of recombination (Takeo et al., 2011; Tanneti et al., 2011).

In nematode oocytes, chromosomes initiate SC formation at the end of the chromosome where the pairing center resides, and rapidly and irreversibly complete the SC (Rog and Dernburg, 2015). The rate of SC assembly is 150 nm/min. The nematode’s six chromosomes initiate synapsis independently and stochastically, completing synapsis within 5 hours as nuclei pass through the transition zone. Movements of the chromosomes by dynein aid the extension of the SC, since Sun mutants that reduce dynein-directed chromosome motion cause a severe reduction in the rate of assembly (34 nm/min). Since *C. elegans* is the only organism so far in which SC kinetics have been measured, the question remains whether SC kinetics show similar behavior in organisms such as yeast and humans, which rely on recombination for synapsis to occur.

In budding yeast, it is difficult to visualize SC kinetics due to the large number of chromosomes in a small nucleus; there are 16 pairs of chromosomes in a ∼2.0 μm diameter nucleus. Fission yeast has only three chromosomes, but fission yeast does not form SC (Bähler et al., 1993). However, the reduced number of synapsed chromosomes in the *zip3Δ* mutant in budding yeast could allow the tracking of SC kinetics. Zip3 is an E3 ubiquitin ligase for which orthologs have been found in many diverse organisms (Agarwal and Roeder, 2000; Jantsch et al., 2004; Chelysheva et al., 2012). Whereas mutants in the *ZIP3* gene in most organisms appear to affect crossover formation, yeast mutants additionally exhibit a reduction in synapsis initiation (Agarwal and Roeder, 2000). When *ZIP3* is deleted, SC initiation occurs predominantly at the centromere and fewer chromosomes form SC (Macqueen and Roeder, 2009).

Here, we take advantage of the reduced number of synapsing chromosomes and initiation sites in the *zip3Δ* mutant to permit the measurement of the real-time kinetics of both assembly and disassembly of the SC on individual chromosomes in yeast. We find that SC assembly in budding yeast occurs by a monotonic increase in length, similar to that observed in *C. elegans*, but that the rate of assembly in yeast is on average about half the rate observed in the nematode. We show that both monophasic and biphasic growth rates are observed, unlike the dynamics in the nematode. The biphasic growth consists of an initial fast rate followed by a slower rate to complete assembly. Final disassembly exhibits a monophasic rate of disassembly. Finally, we uncover a process that we term “abortive SC disassembly” which is distinct from final SC disassembly, in which SCs depolymerize before the cell completes the SC assembly phase. We propose that abortive SC disassembly may represent the dissolution of defective/non-productive or non-homologous SCs. We suggest that this is a mechanism that the cell might employ to correct synapsis or to resolve interlocks before interactions between chromosomes are cemented in place.

## Materials and methods

### Meiotic time course and detection of SCs

For all strains, meiosis was induced by first growing the cells in 2 mL of YPD supplemented with 1.0 mM adenine, and incubating in a roller drum at 30°C for exactly 24 h, then isolating cells by centrifugation and transferring to 10 mL of 2% potassium acetate in 125 mL flasks at 30°C on a platform shaker at 230 rpm. Cells were then harvested at defined time points, and prepared for live microscopy by concentrating harvested cells in sporulation media and then centrifuging them onto a Concanavalin A-treated dish environmental chamber (Bioptechs Inc. # 04200415C, Butler, PA) in the well of a silicone gasket (Grace Bio-Labs #CWCS 50R-1.0, Bend, OR). Cells in the Bioptechs dish were then mounted on an OMX microscope (Dobbie et al., 2011) at 30°C and viewed using a heated objective (×100 Olympus 1.45 NA oil immersion at 30°). Details for live cell imaging can be found in Pollard and Fung (2017).

Zip1-GFP and synapsed chromosomes were detected in a 50 nm window centered at 525 nm using an excitation frequency of 488. The excitation laser was attenuated to 3.5% or 0.86% and individual exposures were 5 ms. Images were acquired in 4 or 10 µm z-stacks with 0.2 µm intervals between sections. The post-acquisition processing of imaged nuclei involved concatenation of all time points, denoising (Boulanger et al., 2010), and deconvolution. Image screening and manipulation, as well as the quantitation of Zip1-GFP signal and the measurement of synaptonemal complex lengths, were performed using PRIISM software (Chen et al., 1992). Automated SC tracing and kinetic measurements were performed using scripts written with MATLAB (Mathworks, Natick MA), although manual tracing of SCs was also performed in PRIISM. 2D projections are either overlaid maximum and summation (max-sum) projections in Z (axial dimension of microscopy) or triple overlays, in which an additional overlay was made with an inverted background and scaled differentially in order to display the nuclear boundary defining the diffuse Zip1-GFP in blue.

### Chromosome spreads, FISH and immunostaining

Chromosome spreads were performed as described previously (Fung et al., 2004). Fluorescent *in situ* hybridization (FISH) was carried out using two adjacent interval-specific DNA probes. For the *LEU2-MAT* interval, the plasmid 12B (Newlon et al., 1991) containing a 20-kb region of chromosome III extending ∼5 kb centromere-distal and ∼15 kb centromere-proximal of the *RPS14A* gene was used to make probe. For the *HIS4-LEU2* interval, a 15-kb region starting at *HIS4* and ending in the middle of *KCC4* was PCR-amplified from genomic DNA in 2-kb segments. Probes were labeled with biotin-14-dATP (Invitrogen # 19524016, Waltham, MA) or digoxygenin-11-dUTP (Roche #11093088910, Basel, CH) and hybridization was performed as described in Dernburg and Sedat (Dernburg and Sedat, 1998). Slides were stained with anti-rabbit Zip1 antibody and then with secondary antibodies: rhodamine anti-DIG and FITC-streptavidin and Cy5 anti-rabbit antibody. To stain DNA, 1 μg/mL DAPI was added to the mount made from 0.1% p-phenylenediamine (Sigma Aldrich #P6001, Burlington, MA) in glycerol. Zip1 polyclonal antibodies were generously provided by G.S. Roeder (Yale University).

### Sporulation frequency

Log-phase cultures in YPAD were transferred to 10 mL of 2% potassium acetate and then shaken in a flask at 30° for 5 days. Cell samples were then prepared on slides and visualized with 3D bright-field microscopy on the OMX microscope. The number of spores present in each cell within the bright field image volume was tabulated.

### Spore viability

Diploids were patched to 2% potassium acetate plates and grown at 30°C for 3 days. Tetrads were dissected onto YPD plates. The frequency of viable spores was determined after 3 days of growth at 30°C.

### Model fitting, adjusted R-square and PRESS statistics

Segmented regression and press statistic calculations for assembly, final disassembly and abortive disassembly rates were performed using R. Code was adapted from https://gist.github.com/tomhopper/8c204d978c4a0cbcb8c0 and https://cran.r-project.org/web/packages/segmented/segmented.pdf.

### Calculation of expected frequency of multiple initiation

Based on the Poisson distribution, we can calculate the probability of seeing k number of initiations with an average number of events, *λ*.

$$f\left(k,\lambda \right)=P\left(k\right)=\frac{\lambda^{k}e^{-\lambda }}{k!}$$

The frequency of seeing ≥ 2 initiations is f ( ≥ 2) = 1−ƒ(0)−ƒ(1). Using a binomial test calculator, the probability of not seeing ≥ 2 initiations after n number of observations can be calculated using n; f ( ≥ 2).

## Results

### *In vivo* visualization of single chromosome synapsis in *zip3Δ* during meiosis

**FIGURE 1:**
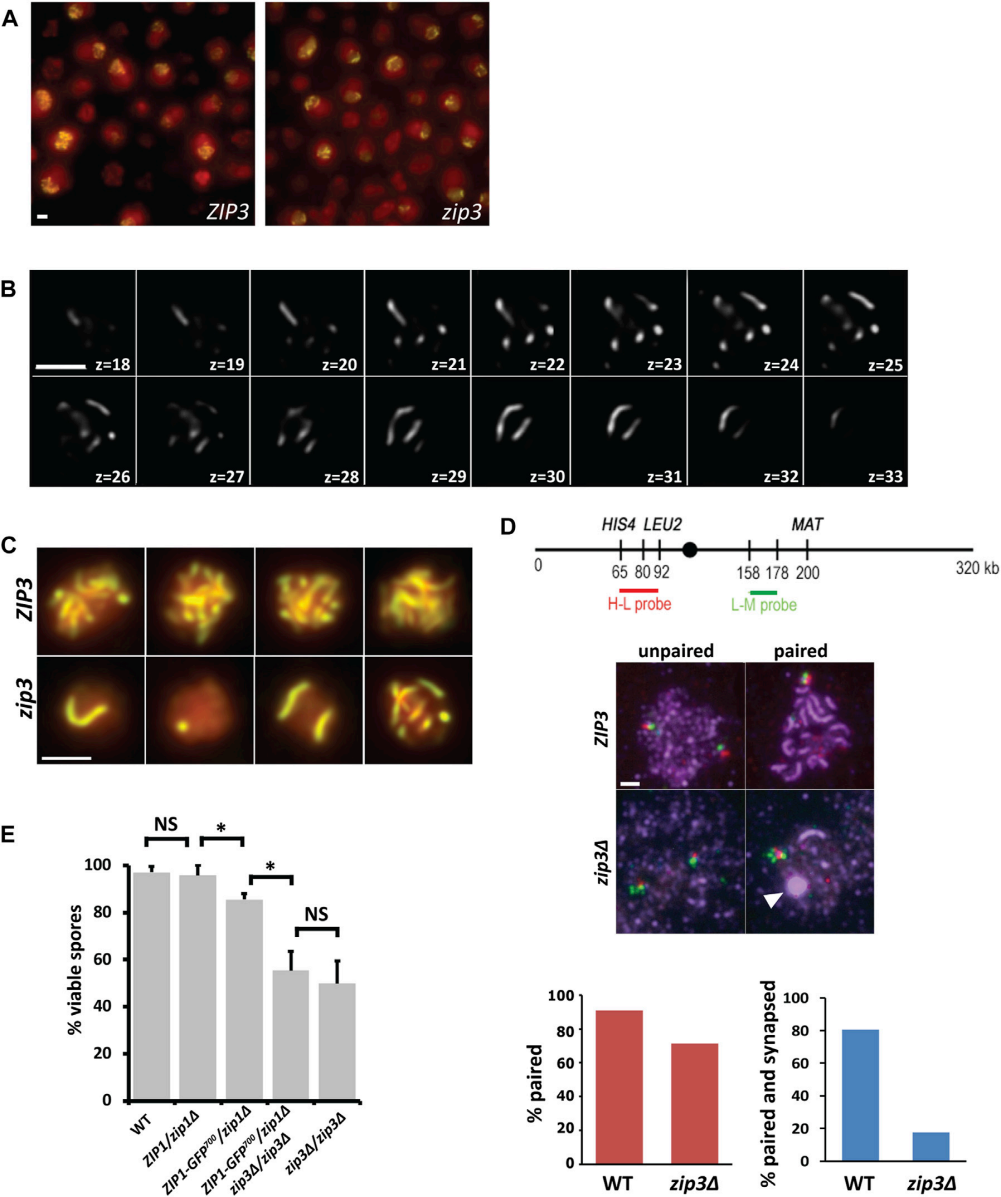
*zip3Δ* improves visualization of synapsis. **(A)** Example of a typical acquisition field of WT (left panel) and *zip3Δ* (right panel) *in vivo* yeast cells expressing Zip1-GFP undergoing meiosis (14 h after meiotic induction) shown as a 2D max-sum projection. Zip1-GFP on synapsed chromosomes is shown in yellow. Nuclei are defined by overall nuclear Zip1-GFP signal shown in red. These images are not for quantitative intensity comparisons. **(B)** z-slices every 0.2 μm from 3D image stack of a nucleus containing 9 Zip1-GFP SCs in a zip3. mutant after denoising and deconvolution. **(C)** 2D max-sum projections of pachytene nuclei expressing Zip1-GFP in ZIP3 (top panel) compared to the equivalent stage in zip3. (bottom panel). Scale bar―2 μm. **(D)** Top panel. Map of FISH probes made to the *HIS4-LEU2* region (H-L probe) and to the *LEU2-MAT* region (L-M probe) on chromosome III. Middle panel. WT and *zip3Δ* pachytene chromosome spreads hybridized with H-L probes (red) and L-M probes (green) and stained with anti-Zip1 antibodies (purple) for WT and *zip3Δ* (middle panel). The white arrowhead indicates a polycomplex. Scale bar 2 µm. Bottom Panel. Red graph shows the percent of HL and LM FISH probes colocalizing for WT (*n* = 33) and *zip3Δ* (*n* = 40). The blue graph shows percent of colocalized probes that are within synapsed regions for WT and *zip3Δ*. **(E)** Histogram of the percent of viable spores for each genotype. For each genotype, between 120–170 tetrads were dissected. A z-test for proportions was used to test for significance (*). Z = 5.64, *p* < 0.00001 between *ZIP1/zip1Δ* and *ZIP1-GFP/zip1Δ*. Z = 5.62 *p* < 0.00001 between *ZIP1-GFP/zip1Δ* and *ZIP1-GFP/zip1Δ zip3Δ/zip3Δ.* NS—not significant. Error bars—STD.

To visualize synapsis of chromosomes in yeast, we performed three-dimensional (3D) time-lapse studies of the synapsis protein Zip1 fused to GFP (Zip1-GFP^700^) in a meiosis-proficient diploid yeast strain (BR 1919-8B) (see Materials and Methods). We constructed a *zip3Δ* mutation in this background to assess SC assembly and disassembly kinetics for individual chromosomes more easily (Figure 1). In *zip3Δ*, the maximal number of synapsed chromosomes attained varies from 0 to 16 chromosomes (Agarwal and Roeder, 2000; MacQueen and Roeder, 2009) (Figures 1A,B). This aspect of the *zip3Δ* mutant strain allows us to monitor synapsis kinetics in nuclei containing only one or two synapsing chromosomes (Figure 1C). Additionally, the use of the *zip3Δ* mutant reduces the complication of interpreting synapsis that normally would start at multiple sites along the chromosome, since ∼85% of synapsis initiates exclusively from centromeres (Macqueen and Roeder, 2009). Despite the reduced extent of synapsis in *zip3Δ*, a high level of pairing is achieved, as measured using a pair of adjacent FISH loci on chromosome III in pachytene chromosome spreads (Figure 1D). These results agree with a prior study of centromere-associated lacO pairing in *zip3Δ* (Voelkel-Meiman et al., 2019). By simultaneously measuring pairing (association of both FISH loci) and synapsis (Zip1 immunofluorescence along chromosomes), we found that the paired loci were only associated with synapsis 17.5% of the time in *zip3Δ* compared to WT (80%) (Figure 1D). Together, these results suggest that a high level of pairing does not ensure high levels of synapsis, and conversely that synapsis is not necessary for high levels of pairing. This ability to align without subsequent synapsis likely contributes to the relatively high spore viability of the *zip3Δ* mutant (50%–58%, ((Macqueen and Roeder, 2009), Figure 1E).

As seen in Figure 1, the *zip3Δ* mutant often forms a polycomplex during pachytene (Figure 1D, last panel, white arrowhead). In the BR background, polycomplexes are aggregates of synapsis-associated proteins that form when the stoichiometry of SC proteins is disrupted, as in the case of various meiotic mutants or with altered expression of meiotic proteins (Sym and Roeder, 1995; Chua and Roeder, 1998). Other organisms and other yeast strains such as SK1 may form polycomplexes in the context of wild-type meiosis, either as a prelude to SC formation or as SCs dissolve (Hughes and Hawley, 2020). The formation of SC is difficult to visualize quantitatively when two copies of the *ZIP1-GFP* ^*700*^ allele reside in a *zip3Δ* background, since the polycomplex is about five times brighter than the synapsing chromosomes. By incorporating a single copy of *ZIP1-GFP* ^*700*^ into a *zip3Δ* diploid whose endogenous copies of *ZIP1* are deleted, the frequency of polycomplex formation was reduced to only 2.2% of nuclei compared to 100% when both copies of *ZIP1-GFP* ^*700*^ are present. In the BR background, only a small difference in spore viability is seen when using hemizygous *ZIP1-GFP* ^*700*^ (85%) in place of hemizygous *ZIP1* (96%). No difference in spore viability is observed between *zip3Δ* strains containing either *ZIP1* allele (Figure 1E), suggesting that replacing *ZIP1* with *ZIP1-GFP* and reducing the copy number of *ZIP1-GFP* has only a minor impact on meiosis. With these strain modifications in place, kinetic measurements of individual SCs are feasible.

### Normal chromosome motion during pachytene exhibited in a *zip3Δ* mutant

**FIGURE 2:**
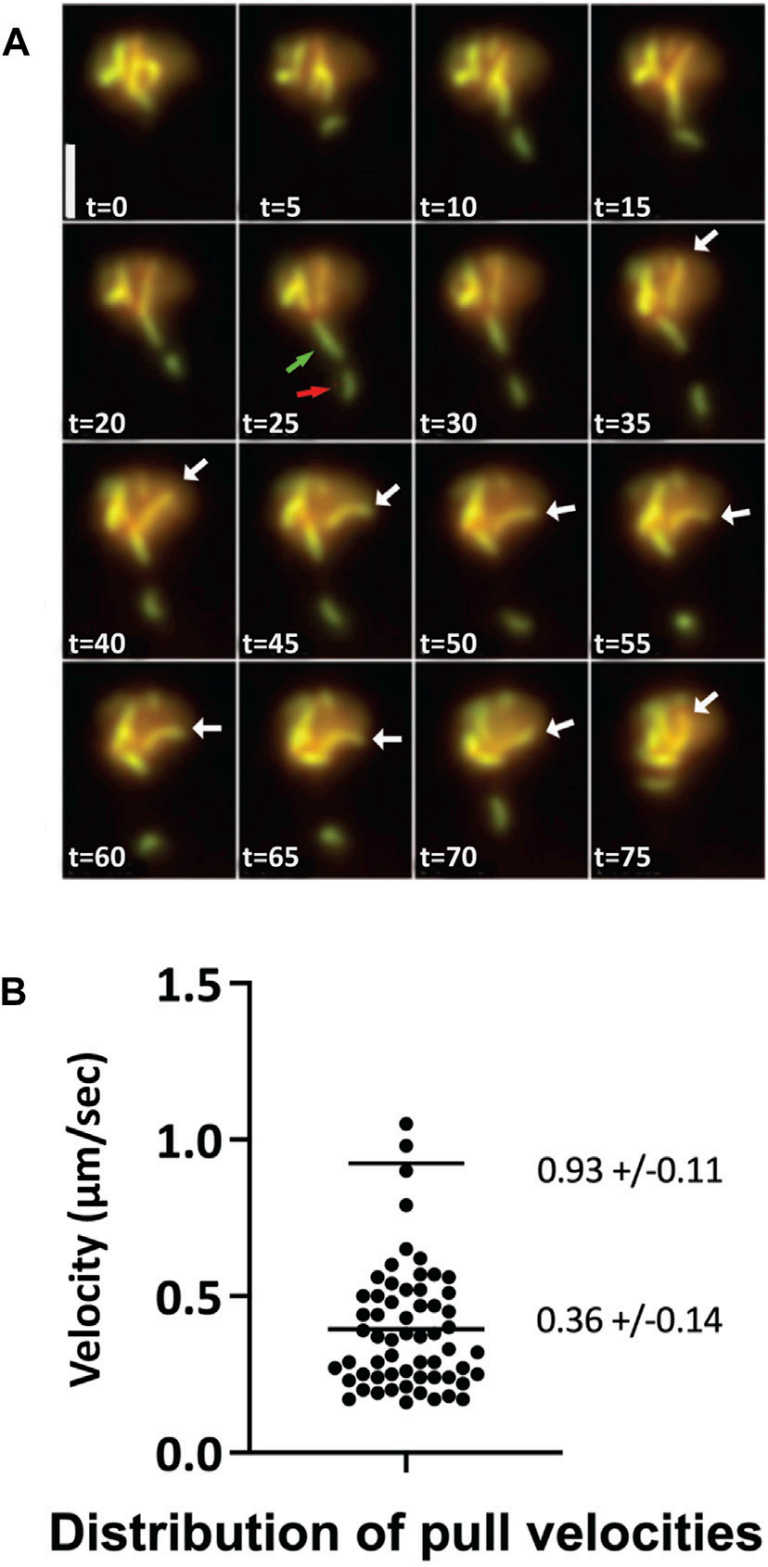
Chromosome dynamics and sporulation observed in *zip3Δ* **(A)** Max-sum projections from a 3-D time series at 5 s intervals of a *zip3Δ* cell expressing Zip1-GFP during pachytene. An example of telomere-led chromosome motion is illustrated by a single long chromosome moving between *t* = 35 s to *t* = 75 s (white arrow). Maverick chromosomes (green and red arrows). Scale bar—2 µm. **(B)** Distribution of velocity measurements for telomere-led motions of pachytene chromosomes from nuclei expressing Zip1-GFP in *zip3Δ*. Averages and STDs are given for the low and high velocity clusters. N = 60.

Meiotic chromosomes undergo rapid, large-scale motions whose function is important in attaining proper and timely homologous alignment (Conrad et al., 2008; Koszul et al., 2008; Navarro et al., 2022). In *C. elegans*, the disruption of this motion in a Sun mutant leads to perturbed pairing and synapsis elongation (Sato et al., 2009; Rog and Dernburg, 2015). Therefore, it is important to establish whether the behavior of chromosomes in *zip3∆* yeast cells resembles the motion of chromosomes observed in wild type. Koszul et al. (2008) characterized the motion of fully synapsed chromosomes marked with Zip1-GFP in permeabilized cells and *in vivo* pachytene nuclei. They observed dramatic movements of chromosomes during the pachytene stage of meiosis mediated by attachment of the chromosomes to actin cables proximal to the nuclear envelope. These telomere-led movements exhibit velocities of 0.3–0.5 μm/s (up to 0.8 μm/s) and are characterized by abrupt transitions of increased velocity. We observe comparable motion in *zip3Δ* (Figure 2A, movies Supplementary Figures S1–S3) with average motion in the 0.2–0.6 μm/s range and higher transitions up to 1.0 μm/s (Figure 2B). Our measurements in *zip3Δ* also agree with the results reported in wild-type cells by (Conrad et al., 2008), who examined the rapid prophase motion by imaging *lacO*-marked regions of the chromosome. Another characteristic of wild-type chromosome behavior in pachytene nuclei, also observed in *zip3Δ* cells, is the presence of “maverick” chromosomes (White et al., 2004). Occasionally, maverick chromosomes are observed to protrude out at a great distance from the bulk of the chromosomes, often with end-to-end chromosome connections in which chromosomes resemble sausages on a string. Figure 2A highlights an example of a time course projection showing both types of behavior, telomere-led motion and maverick chromosomes, in the *zip3∆* mutant within a single nucleus. Overall, prophase chromosome movement in *zip3∆* appears to be comparable to that previously observed in wild type.

### Optimization of conditions for *in vivo* microscopy

**FIGURE 3:**
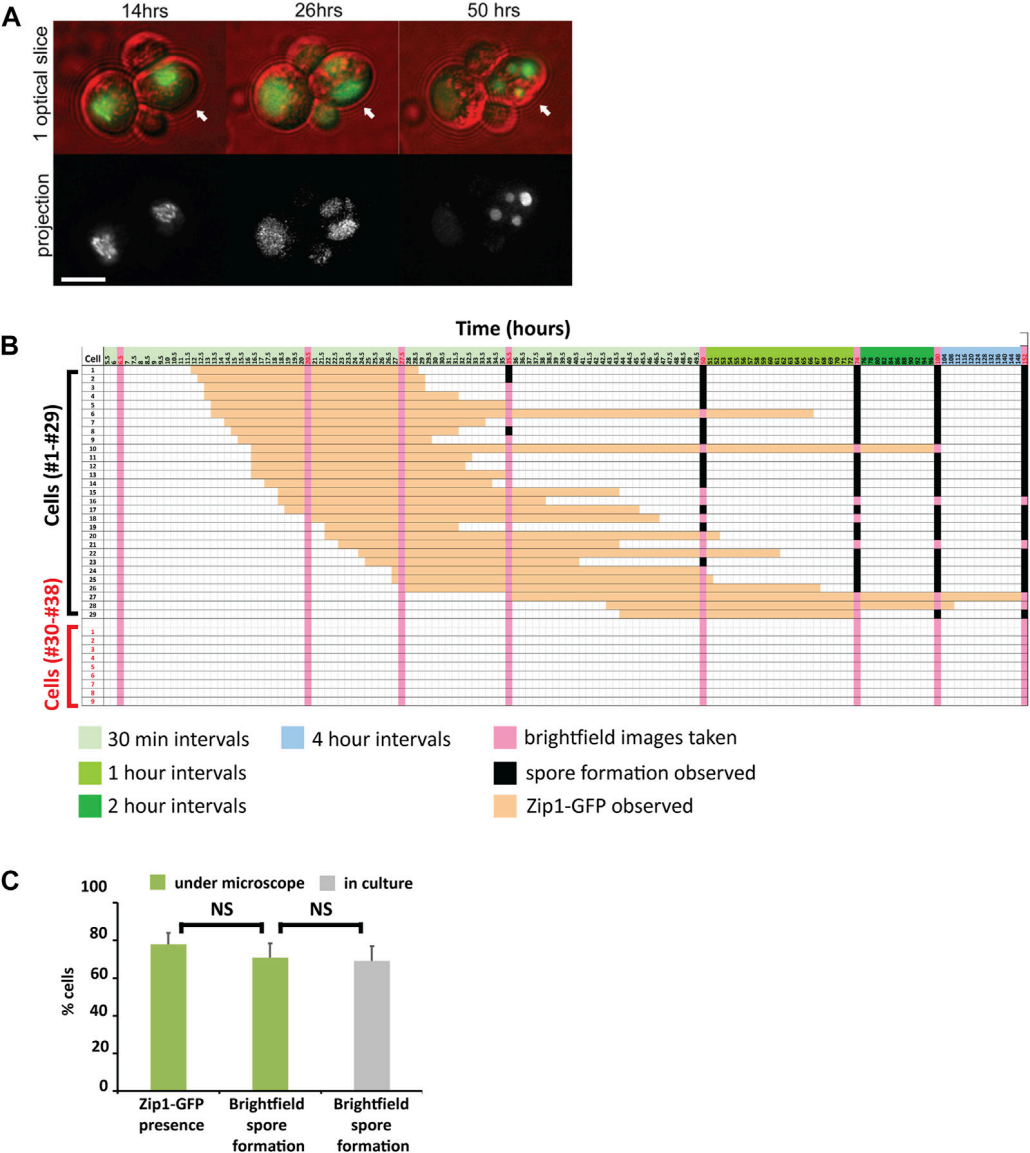
Imaging conditions do not perturb meiotic progression **(A)** Examples of Zip1-GFP and spore formation examples are shown at three timepoints as meiosis progresses in live cells. Zip1-GFP signal (green) is overlayed by brightfield to detect cell and spores (red). Both single optical slice and 2D projections are shown. Although 3 cells show Zip1-GFP expression during the time course, only one progresses to spore formation. Scale bar 5 µm. **(B)** Cells were tracked for meiotic progression for 152 h to determine the frequency of cells that enter meiosis as well as the frequency of cells that eventually form spores, to determine if the imaging conditions perturbed sporulation. A representative experiment in which 38 cells were tracked, of which 29 entered meiosis (cells with black numbers, first column) as determined by Zip1-GFP detection (orange squares) and 9 (cells with red numbers) did not. Cells were imaged for Zip-GFP initially at 30-minute intervals until 50 h (pale green, top column headers), then 1-hour intervals until 74 h (olive green), then 2-hour intervals until 100 h (dark green), followed by 4-hour intervals until 152 h (blue). At 6.5, 20.5, 27.5, 35.5, 50, 74, 100, and 152 h, brightfield images were acquired to assess for spore formation (pink columns). Black boxes indicate when spores were detected. **(C)** The number of cells entering meiosis and the number of cells forming spores are compared. Cells were counted if any spores (1–4) were observed. The average percentages were determined from five experiments performed as described in **(B)** above. A total of 179 cells were tracked. Experiments performed under the microscope using our optimized imaging conditions are shown in green. The frequency of sporulation was also calculated by counting spore formation in brightfield for comparison (gray) after 5 days of normal culturing in flasks.

It is essential for *in vivo* microscopy studies to demonstrate that the imaging conditions do not perturb the event of interest and subsequent cellular progression (Carlton et al., 2010). Our protocol ensures that cells can complete meiosis without exhibiting phototoxic effects (Supplementary Figure S4). To document whether our imaging conditions permit completion of meiosis, cells were continuously imaged to determine whether they formed spores (Figure 3A). We used fluorescence microscopy to collect 3D optical sections (Figure 1B) of wild-type Zip1-GFP strains starting at 8 h after meiotic induction through early zygotene (∼12–16 h) when synapsis is initiating, through pachytene (∼16–21 h), when synapsis is complete and then at greater time intervals up to 152 h to determine further meiotic progression (Figure 3B). Spore formation was monitored by brightfield microscopy at various times throughout the time course (Figure 3B, pink vertical columns). For the *ZIP3* strain, on average 77.8% ±0.6 SD (*n* = 179, 5 experiments) of the cells enter meiosis, based on number of cells expressing Zip1-GFP. The overall sporulation frequency (69%) observed under our imaging conditions was equivalent to the sporulation frequency measured under normal sporulation conditions in culture (69%) (Figure 3C). Thus, the ability to sporulate is not affected by the imaging conditions, suggesting that photodamage is minimal.

### Reduced Zip1 expression and delayed synapsis kinetics in the *zip3Δ* mutant

**FIGURE 4:**
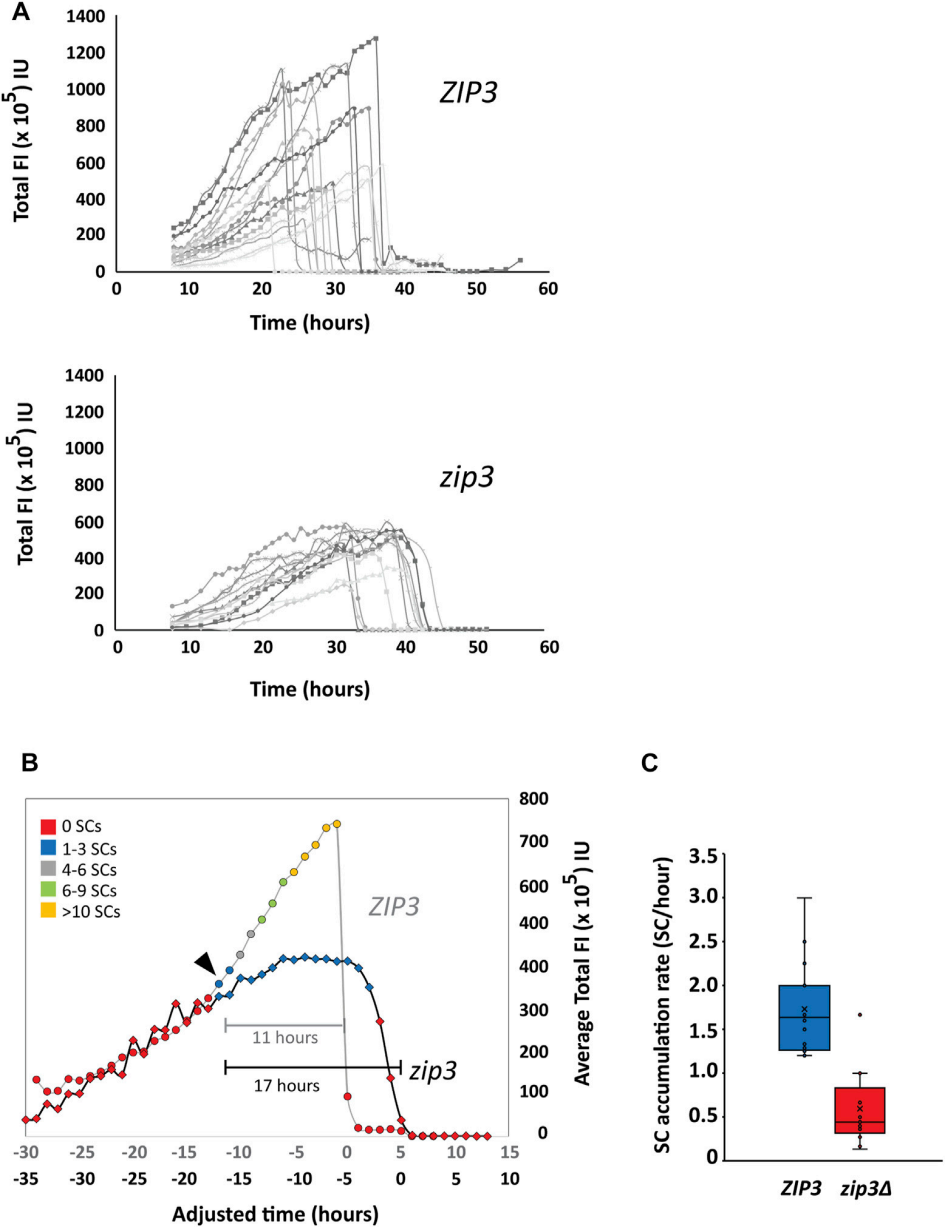
Quantitation of Zip1 levels. **(A)** Top Panel. Profiles of Zip1 levels from individual time courses of *ZIP3* cells expressing Zip1-GFP. *n* = 16.3D optical sections of Zip1-GFP in sporulating strains were acquired for 50 h at 1-h intervals starting at 8 h after induction of sporulation. Fluorescence intensity (FI) was measured as a proxy for Zip1 levels at each time point in arbitrary intensity units (IU). Bottom Panel. Profiles of Zip1 levels from individual time courses of *zip3Δ* cells expressing Zip1-GFP. *n* = 13. **(B)** A comparison of average total Zip1 FI levels during prophase in *ZIP3* (circles) vs. *zip3Δ* (diamonds) strains. Given the asynchrony of synapsis initiation times, individual time courses for *ZIP3* (*n* = 16) and *zip3Δ* (*n* = 13) were aligned such that time zero represented the time at which Zip1-GFP was depleted. The time points were adjusted relative to time zero and average Zip1 levels were calculated for each time point for both strains. These profiles were then aligned to each other based on the time synapsis is first detected. Two adjusted time axes are presented for *ZIP3* (grey) and *zip3*Δ (black). The general number of SCs is distinguished with colored markers: Red—0, Blue—1–3, Gray—4–6, Green—7–9, and Orange—>10 synapsed regions. The black arrow indicates the Zip1 threshold at which synapsis is first observed. Brackets shows length of time for synapsis for *ZIP3* (gray) and *zip3*Δ (black). **(C)** The rate of SC accumulation for *ZIP3* (blue) and *zip3*Δ (red). The rate was calculated by calculating the time it took to first reach the maximum number of distinguishable SCs.

Zip1 expression was monitored over the course of prophase to determine the relationship between Zip1 expression and SC assembly. To measure Zip1 expression *via* fluorescence intensity (FI), 3-D optical sections of Zip1-GFP in sporulating strains were acquired for 50 h at 1-h intervals (Materials and Methods). We measured the total nuclear FI of Zip1-GFP and the volume for each nucleus to assess the amount of Zip1-GFP at each time point. Profiles of total nuclear Zip1 Fl for several individual cells over the course of prophase I are shown for *ZIP3* and *zip3Δ* (Figure 4A). We observe a large variation in the duration of Zip1 presence for both *ZIP3* and *zip3Δ* in individual cells.

In order to compare Zip1 profiles in wild type and *zip3Δ* cells that start accumulating Zip1-GFP at different times (Figure 4A), we aligned each profile by setting time to zero when Zip1-GFP is first depleted for both *ZIP3* and *zip3Δ*. The average total Zip1-GFP FI and number of SCs for the time courses were then calculated and these two averaged profiles were aligned to each other at the point at which synapsis initiates (Figure 4B, arrowhead). We find that Zip1 expression initially increases before the first SC appears and continues to rise both for *ZIP3* and *zip3Δ*. Before Zip1 levels decline at the end of pachytene, Zip1 expression plateaus in *ZIP3* for 1.5 h on average, while this period lasts ∼4 times longer (6.3 h) for *zip3Δ* (Figure 4B). We also observe a difference in the maximum Zip1 intensity achieved for *ZIP3* (740 FU ± 66 SE) as compared to *zip3Δ* (424 FU ± 29 SE) which only attains 57% of wild-type levels. The abrupt degradation of Zip1 at the end of pachytene occurs within 1 hour in *ZIP3* wild-type cells, which is much faster than that observed for *zip3Δ* mutants, which on average occurs over ∼3 h. Overall, this leads to an average 6-h greater duration of synapsis for *zip3Δ* (17 h) than for *ZIP3* (11 h) (Figure 4B).

### SC formation occurs after equivalent Zip1 levels are reached

We observe that SCs first appear when the average total Zip1 FI reaches about 380 × 10^5^ FI for wild type and similarly to 310 × 10^5^ FI for *zip3∆* (Figure 4B, black arrowhead). This suggests that synapsis initiation may require a threshold level of Zip1 concentration. However, we cannot distinguish at this point whether it is a threshold concentration of Zip1 or the stage of meiotic progression that is permissive for SC initiation. Like Zip1 production, the rate of accumulation of SCs is faster for wild type (1.7 ± 0.5 SD synapsed chromosomes/hour) than for *zip3∆* (0.6 ± 0.4 SD synapsed chromosomes/hour) synapsed chromosomes/hr) during this period (Figure 4C). For wild type, the maximal number of SCs could not be accurately counted but was determined to be greater than ten. A previous study using fixed nuclear spreads showed an average of five synapsed chromosomes in *zip3∆* compared to the 16 expected in wild type (MacQueen and Roeder, 2009). From Figure 4B, it appears the number of SCs peaks at 1–3 SCs in *zip3∆.* This discrepancy is likely due to the inability to accurately count nuclei with 10–16 SCs in intact cells as compared to chromosome spreads as well as the lower number of nuclei used to determine the average in Figure 4A.

### SC assembles continuously with either monophasic or biphasic kinetics in *zip3Δ*

**FIGURE 5:**
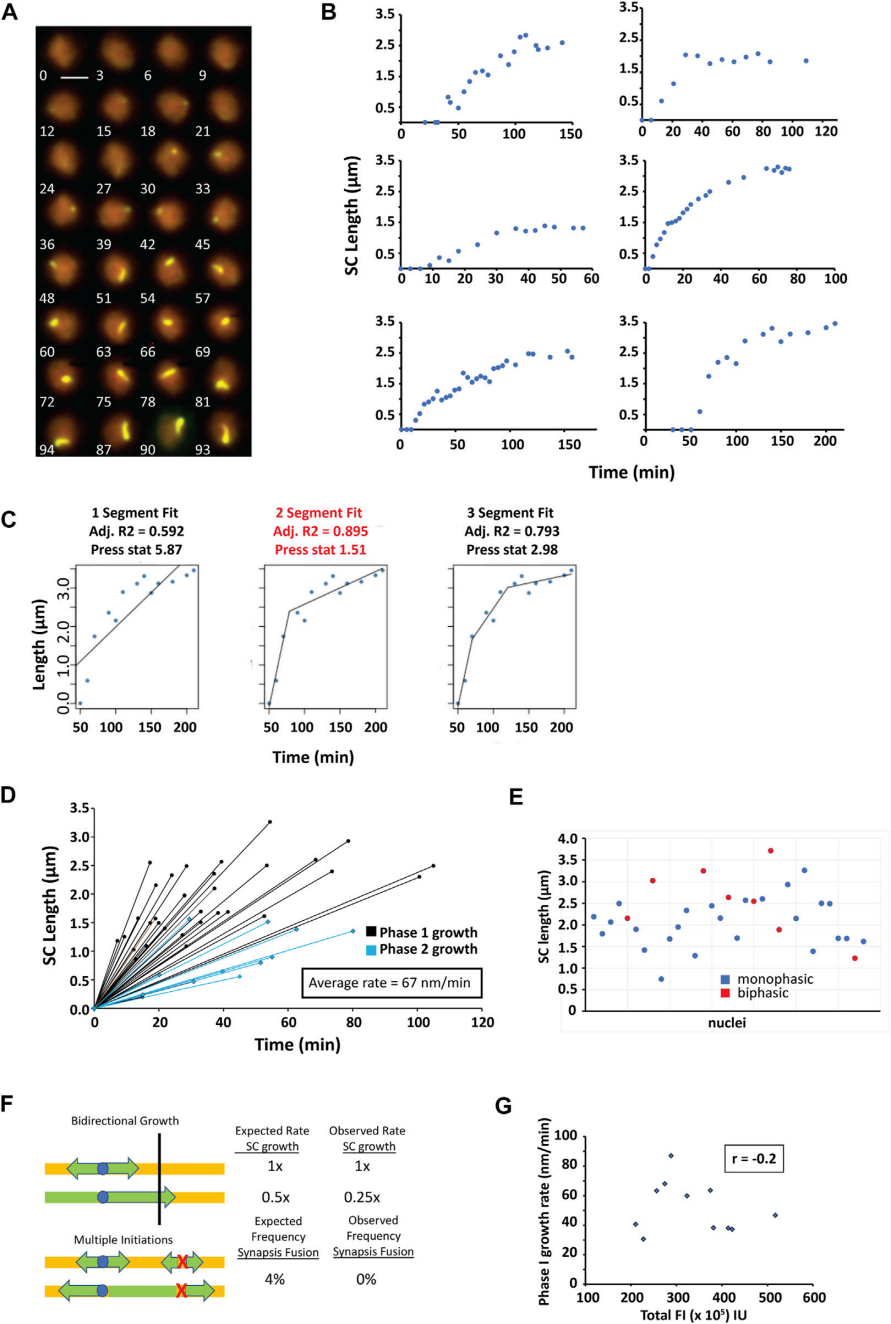
Synapsis assembly. **(A)** A time series of 2D max-sum projections at 3-min intervals from 3D image stacks of a single nucleus undergoing synapsis assembly visualized by Zip1-GFP. Scale bar—2 µm. **(B)** Six representative nuclei containing a single chromosome for which SC length was measured as a function of time during SC assembly. **(C)** Segmented regression was applied to the assembly data using 1–3 segments. The best fit model was determined using the adjusted R2 (adj. R2) and cross-validated with the PRESS statistic. Best fit models have the highest adjusted R2 and lowest PRESS statistic. In this example, the 2-segment fit (text highlighted in red) represents the best model. **(D)** Both monophasic and biphasic models were found for SC assembly. The initial rate of SC growth for both monophasic and biphasic models are shown illustrated as black lines with round markers. For the biphasic models, the second SC growth rate is shown as blue lines with diamond markers. **(E)** Total SC length for all nuclei with monophasic and biphasic growth indicated (blue circles–monophasic**,** red circles–biphasic) **(F)** A model in which SC growth initiates bidirectionally from an acrocentric centromere. A faster bidirectional (initial) SC assembly rate is predicted to slow to 50% of the initial rate once the shorter end is reached (top panel). Predicted and observed rates are shown. Expected and observed detection of individual synapsis initiation sites fusing into one synapsed chromosome are shown (bottom panel). **(G)** Plot of the phase 1 rate vs. total Zip1-GFP FI when the appearance the first SC is observed. r is calculated correlation coefficient. A subset of the data was used due to only a few data sets had intensities associated with the growth under the exact same conditions.

In *zip3∆,* it is possible to observe and measure individual SCs assembling from initiation, through elongation, to completion of synapsis (Figure 5). Quantitation of synapsis elongation rates is greatly facilitated by the tracking of cells when no other synapsed chromosomes are present. In live yeast, chromosomes range from less than 0.5 µm to over 3 µm in length. To obtain enough measurements during elongation, our analysis focused on nuclei in which only a single long chromosome synapsed (∼0.14% of observed nuclei). Cells were imaged at intervals ranging from 3 to 10 min between each 3-D stack (shown as 2-D projections in Figure 5A) with most examples at 3-minute intervals. To determine whether the observed SC elongation represents continuous, discontinuous and/or step-wise assembly, we plotted the length of the synapsed region over time (Figure 5B). Segmented regression was used to determine whether SC assembly occurred at single or at multiple rates (Figure 5C). A predicted R-squared was calculated and cross-validated with the predicted residual error sum of squares (PRESS) statistic to distinguish the best model to minimize overfitting (Alcantara et al., 2022). Logarithmic fits were also performed, but the average R^2^ compared to R^2^ obtained from the segmented regression was worse (0.84 vs. 0.98).

In 34 SC assembly events measured, 65% of the assembly was monophasic and the rest (35%) showed biphasic assembly. In all cases, SC assembly monotonically increased, as no long pauses between steps were observed. The average assembly rate was 67 nm/min (range from 12 to 165 nm/min, Figure 5D), which is about half the rate seen for SC assembly in *C. elegans*. The average monophasic growth (56 ± 23 SD nm/min) is significantly slower than that observed for the first and faster part of biphasic growth (88 ± 42 SD nm/min). For all biphasic SC growth, the second phase of SC assembly (Figure 5D, blue lines) is slower (19 ± 12 nm/min P_t. test_ = 0.002) than the first phase. For biphasic growth, the first rate of growth contributes on average to 59% ± 16% (SD) of the final SC length. SC lengths for each assembly event can be found in Supplementary Table S2 and Figure 5E.

### *zip3Δ* chromosomes synapse from a single initiation

In *zip3* mutants, there are fewer initiation events, of which 85% of the SC initiations come from centromeres (Macqueen and Roeder, 2009). In wild type, synapsis initiation occur at centromeres but more frequently at recombination-associated sites, and multiple initiations are observed on each chromosome (Tsubouchi et al., 2008). Because we did not observe multiple initiations in the 230 SC assembly events monitored, we wanted to assess whether this could be attributed to an insufficient number of observations. Based on Poisson statistics and the average number of SCs in a *zip3Δ*, we can calculate the likelihood of seeing multiple initiations on the same chromosome (see Materials and Methods). Given that there are five SCs on average in *zip3Δ*, we would expect to see two or more initiation sites occurring on the same chromosome ∼4% of the time. In the 230 SC assembly events that we observed, we see no instances of multiple nucleation events which would be detected by the fusion of elongating SC stretches (Figure 5F, bottom panel). Since the binomial equation predicts that there is a 0.01 percent chance of missing such an event in the 230 SC assemblies observed, this suggests that Zip1 initiates only from one nucleation site in *zip3Δ*.

### Potential models for biphasic growth

The longest SCs we measured were ∼3 µm long and likely correspond to full length chromosome IV SCs since the next longest chromosome, chromosome XV is estimated to be ∼2.3 µm and thus would not be mistaken for chromosome IV. The fact that only one initiation site is used for synapsis in *zip3∆* implies that the same initiation site is used twice: in opposite directions to complete synapsis on that chromosome. Thus, we consider that the synapsis is bidirectional, although the two initiations may not be simultaneous. One model to explain biphasic growth could be the result of off-center centromeres (i.e., neither acrocentric nor metacentric) as in chromosome IV that initiates synapsis from the same site bidirectionally without much delay between initiations (Figure 5F, top panel). In this scenario we expect that for biphasic SC assembly, the second phase of growth would occur when one end of the SC reaches the end of the chromosome, such that only the other end continues to grow with a second phase growth rate 50% of the initial rate (Figure 5F, top panel). Instead, the second phase growth rate was 25% of the initial rate, significantly lower than expected (P_t.test_ = 0.002). Another possibility is that nuclear Zip1 concentration influences SC elongation rates given the results that cells with extra copies of *ZIP1* synapse earlier (Voelkel-Meiman et al., 2012). To test whether changing Zip1 levels influences the elongation rate, we asked whether Zip-GFP total FI correlates with SC elongation rates at the time of appearance of the first SC (Figure 5G). A correlation coefficient *r* = −0.2 was observed indicating no correlation between the starting concentration of Zip1 and SC elongation rate. This suggests that different nuclear concentrations of Zip1 may not be dictating the observed biphasic rates.

### Final disassembly of SCs is accompanied by degradation of Zip1

**FIGURE 6:**
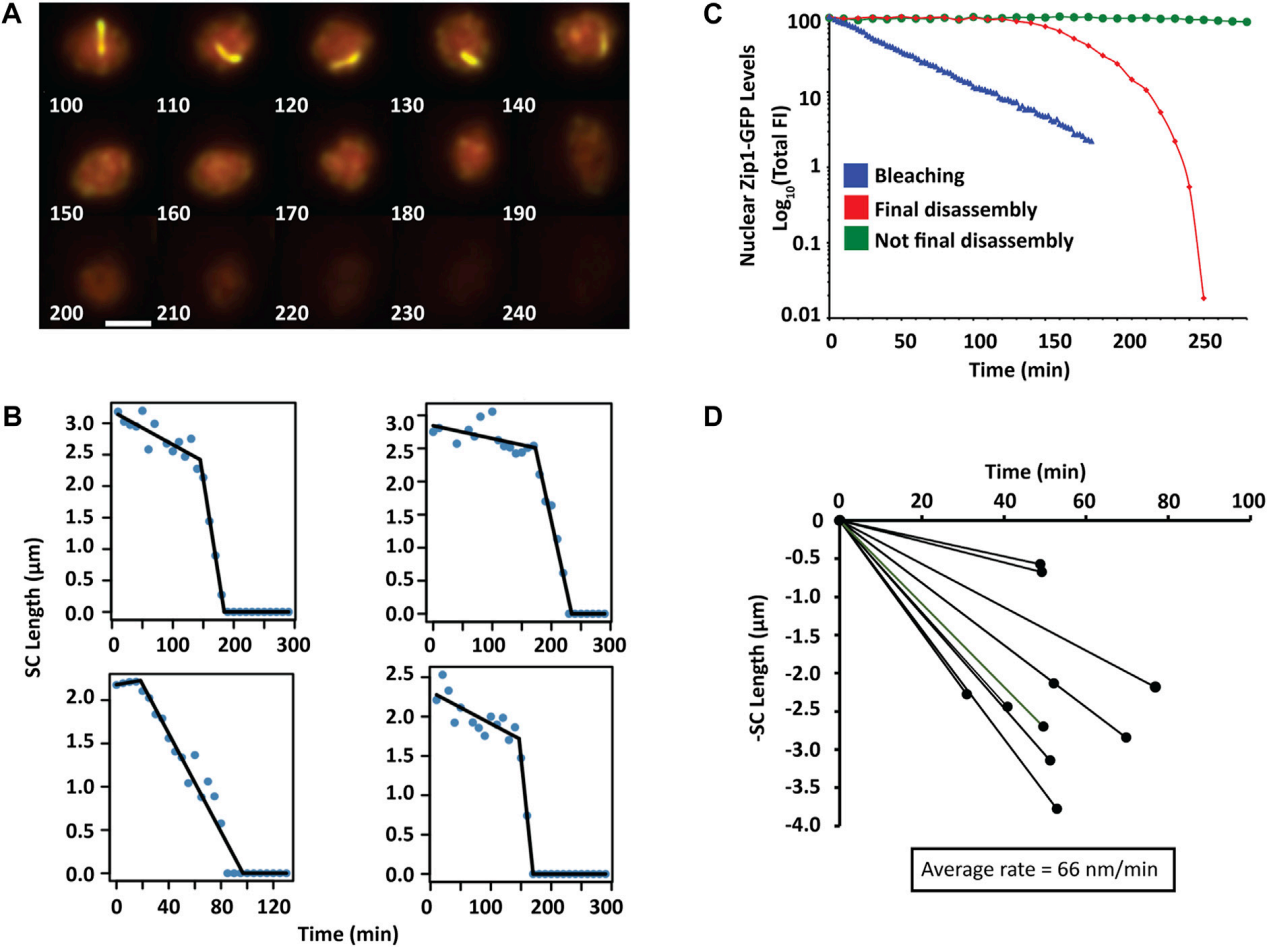
Final SC disassembly **(A)** A time series of 2D max-sum projections at 10-minute intervals from 3D image stacks of a single *zip3Δ* nucleus with a single chromosome undergoing final synapsis disassembly visualized by Zip1-GFP. Scale bar—2 µm. **(B)** Four representative nuclei containing one chromosome for which SC length was measured as a function of time during the final disassembly of the SC. **(C)** Comparison of nuclear Zip1-GFP fluorescence levels when SCs are all disassembling (red diamonds), not at final disassembly (green circles), not disassembling but subjected to heavy bleaching conditions (blue triangles). Log plots are normalized to the respective maximum intensity. **(D)** Disassembly of SC is monophasic. The rate and length of each SC disassembly is plotted (*n* = 10).

Final SC disassembly is accompanied by a rapid decrease in Zip1 levels, such that the majority of Zip1 is removed within 1 hour for *ZIP3* (Figure 4B) and ∼3 h for *zip3∆.* At exit from pachytene, Zip1 is removed from chromosomes with a minor amount of Zip1 protein remaining at the centromeres (Jordan et al., 2009; Newnham et al., 2010). The disassembly of single long chromosomes in *zip3∆* was assessed as in our previous SC assembly measurements*.* Final SC disassembly occurs *via* shortening of the SC from the ends (Figure 6A). We also explored the possibility that SCs were also dismantled at specific foci, similar to foci used for initiation. However, we saw no appearance of gaps within the shortening SCs that would have been indicative that SC were being dismantled at specific internal sites.

To assess the rates of disassembly, we plotted the SC length as a function of time and performed segmented regression to determine whether disassembly was monophasic or occurred at multiple stages (Figure 6B). In all cases, final disassembly was monophasic with an average rate of −66 ± 30 (SD) nm/min, which is similar in magnitude to the initial rate observed for SC elongation. It is possible that the disassembly rate is actually slower than appears since if disassembly occurred simultaneously at the ends, each end would disassemble at half the rate at which the overall length was shortening. In many instances, there was an initial phase that occurred at a very low rate at which SCs were degraded (5 nm/min ± 5 SD). Since this rate was so low, we did not include this period as a separate phase given the error of measurements. This programmed loss of Zip1 is distinguishable from bleaching artifacts (Figure 6C). Figure 6D shows the distribution of SC lengths as a function of time from which the rates were calculated.

### Abortive disassembly occurs during the SC accumulation phase

**FIGURE 7:**
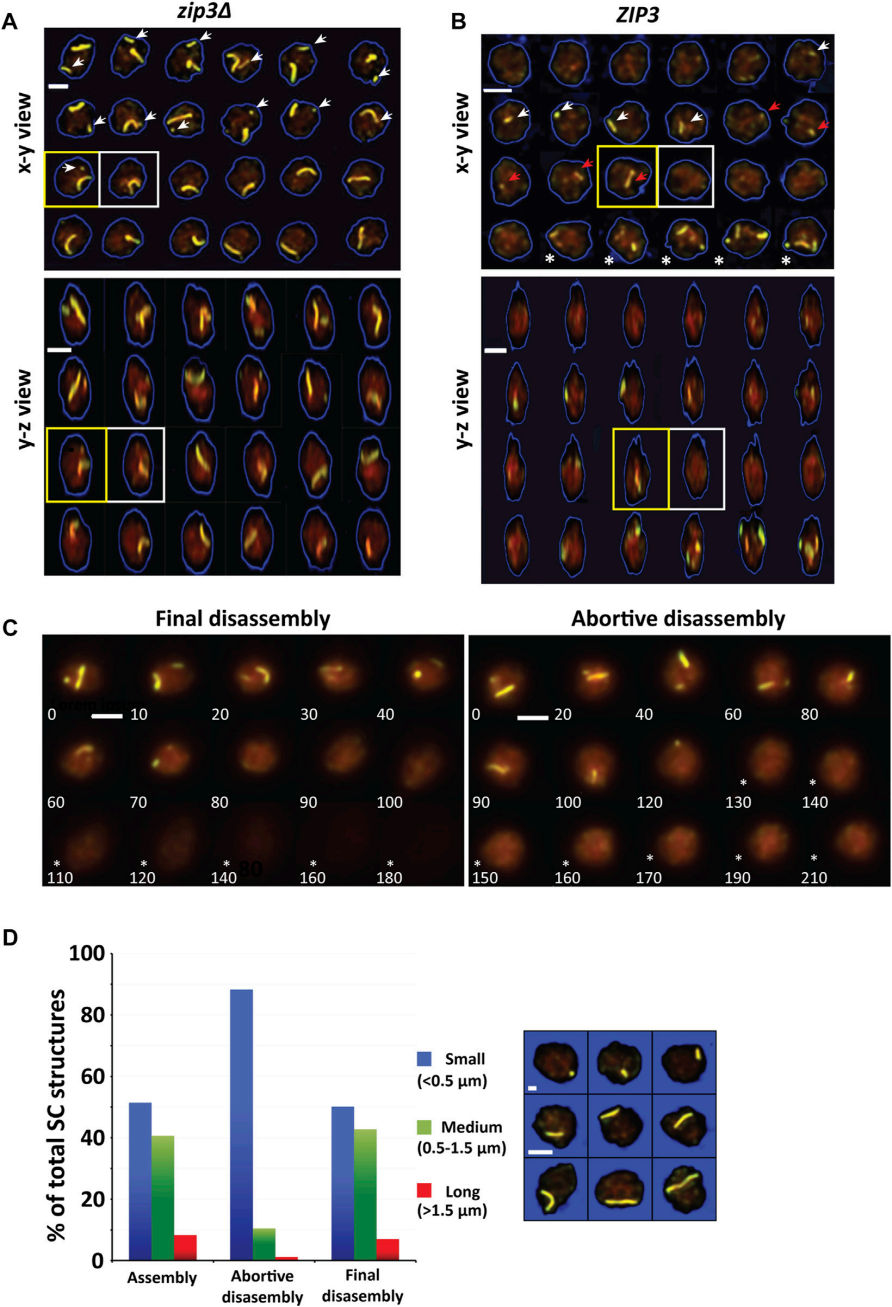
Abortive synapsis disassembly. **(A)** A time-series of 2D triple-overlay projections from 3D image stacks of a single *zip3Δ* nucleus at 5-minute intervals showing abortive disassembly for the smaller (white arrow) of the two chromosomes. The yellow square outlines the last time frame in which the smaller chromosome is last observed. The white square shows time frame in which smaller chromosome is no longer observed. Below is the same time-series in a y-z view. Scale bar—2 µm. **(B)** A time-series of 2D triple-overlay projections from 3D image stacks of a single *ZIP3* nucleus at 20-minute intervals showing abortive disassembly chromosomes. The white arrow indicates a SC that formed and then disassembled. The red arrow represents either the same SC as indicated by the white arrow that did not fully disassemble or an SC that newly formed. The yellow square outlines when the chromosome is last observed. The white square outlines time when chromosome has disassembled. Several timepoints later after a period in which there is no SCs, new SCs form in the timeframes indicated by (*). Below is the same time-series in a y-z view. Scale bar—2 µm. **(C)** Abortive synapsis disassembly is distinguishable from final disassembly by the high levels of Zip1 that remains (right panel, timepoints 130–210 indicated by *) as compared to final disassembly of the SC when Zip1 levels decrease (left panel, timepoints 110–180 indicated by *). Left panel–final disassembly. Right panel–abortive disassembly **(D)** Distribution of SC sizes divided into small (<0.5 µm), medium (0.5–1.5 µm) and large (>1.5 µm) for SC assembly (*n* = 230), abortive disassembly (*n* = 171) and final disassembly (*n* = 530). Images show example SCs belonging to each size class.

While obtaining examples of SC disassembly, numerous cells were found in which the disappearance of an SC is not immediately followed by Zip1 degradation. Indeed, in many of these cases, other SCs persist, and additional SCs continue to form as shown in Figure 7A. Whereas the great majority of examples were obtained from *zip3∆* strains, rare examples were uncovered from *ZIP3* (Figure 7B). These cells have not progressed to the end of prophase, since Zip1 levels remained high and nascent SCs were often still accumulating. We have termed this type of SC disassembly “abortive SC disassembly.” Unlike final disassembly in which Zip1 levels decrease by 50% within an hour and a half (Figure 4B), during abortive disassembly, Zip1 levels remain high well after no SCs are seen (Figure 7C). In a 5-h period, about 30% of nuclei show an instance of abortive disassembly (*n* = 191).

Another difference between final and abortive disassembly is in the size distribution of SCs that are involved in the two processes (Figure 7C). Yeast chromosomes range widely in size from ∼0.5 µm to ∼3 µm with about 25% small (<0.5 microns, as determined from live cells, Figure 7). Whereas the distribution of SC sizes observed for assembly and final disassembly are similar, there is a much stronger bias for small SCs (88%) to be disassembled abortively than during final disassembly (51%) (Figure 7D). Though rare, 15 medium and long SCs experiencing abortive disassembly were characterized for the kinetics of abortive disassembly. Like final disassembly, abortive disassembly is monophasic. However, the average rate for abortive disassembly was much lower 25 ± 1 (SD) nm/min vs. 66 ± 30 (SD) nm/min for final disassembly. Together these results suggest that abortive SC disassembly and final disassembly are distinct.

## Discussion

### Models of biphasic growth

Before this study, the real-time kinetics of synapsis had only been visualized in *C. elegans* (Rog and Dernburg, 2015), an organism that does not rely on recombination to pair or synapse its homologous chromosomes. We set out in this study to examine the kinetics of synapsis in yeast, which is more like humans in that it depends on recombination for its pairing and synapsis. Using a *zip3Δ* mutant in yeast, we were able to unambiguously follow SC assembly and disassembly from a single initiation site on a single chromosome. We saw that SC kinetics in both organisms had distinct differences. In contrast to what has been reported in C*. elegans,* in which the kinetics of SC formation exclusively fit a single rate of elongation, in yeast, we found biphasic elongation 35% of the time. Analysis of synaptic initiation in yeast suggested that synapsis from most centromere-initiated SCs was unidirectional (Tsubouchi et al., 2008). However**,** a single SC initiation site in *zip3Δ* is responsible for synapsis of both arms**,** implying that SC initiation must be bidirectional (i.e., proceed in both directions from a single point). Bidirectional synapsis from a non-centric centromere might account for the two rates of elongation as the short arm completes synapsis earlier, leaving the long arm synapsis to finish alone. However, the magnitude of the rate reduction (greater than the expected two-fold reduction if the elongation rates are equal) suggests that other factors could be contributing as well, such as unequal elongation rates for each side of the initiation site.

Another alternative is a model in which the slower second phase of SC elongation may to be due physical constraints that increase as SC lengths become long. In this case, we would have expected that biphasic growth would be seen more frequently on the longer SCs. Consistent with this, the average SC length is longer for cells exhibiting biphasic growth (Figure 5E).

An intriguing possibility for the observed biphasic growth rate is that the slower rate may be due to chromosome interlocks. Since in yeast, all chromosome ends are embedded in the nuclear membrane, as chromosomes pair, other chromosomes may become trapped and obstruct pairing or alignment in advance of SC formation (Navarro et al., 2022). This would in turn impede synapsis and thereby attenuate the rate of SC assembly in the region of the interlock. It was proposed that entanglements can be resolved by motion of the entrapped chromosome to the telomeric end where the chromosome can escape (Navarro et al., 2022). The delay caused by clearing the entanglement might account for the slower rate of Zip1 assembly. Such a delay might not be seen in *C. elegans*, which is unusual in that only one end of the chromosome is associated with the nuclear envelope, presumably making it easier for interlocks to resolve.

### Yeast SC elongation rate is likely not affected by *zip3Δ* mutation

Overall, the rate of SC formation based on Zip1-GFP images in yeast on average was 67 nm/min which is approximately two-fold slower than the average rate obtained from nematodes (150 nm/m) (Rog and Dernburg, 2015). This raises the possibilities that either synapsis is slower in yeast, or that the *zip3Δ* mutant impairs synapsis elongation rates. While the numbers of SCs are reduced in *zip3Δ* mutants, a full complement of SCs is restored by a mutation in *FPR3* (Macqueen and Roeder, 2009). In the *fpr3 zip3* double-mutant, the inhibition of synapsis initiation at the centromeres is removed, which allows the cells to form SC on all chromosomes. They found that in *fpr3 zip3* the cumulative lengths of SCs per nucleus scored at similar time points were not significantly different from wild type. If elongation rates were slower in the *zip3Δ* mutant than wild type, we would not have expected the *fpr3 zip3* mutants to attain wild-type SC lengths at wild-type rates. However, we cannot fully eliminate the possibility that Fpr3 has some effect on elongation rate. To fully address whether the *zip3Δ* SC elongation rate is representative of the wild-type SC elongation rate, future developments to allow observations of single chromosomes in wild type are needed.

### Factors that might affect overall synapsis period

Factors other than Zip1 assembly rates influence the time it takes to complete synapsis of the entire complement of chromosomes. In some organisms like *Drosophila*, the centromeres are the synapsis initiation sites and they are already paired at the start of meiosis (Takeo et al., 2011; Tanneti et al., 2011). In nematodes, pairing centers present on a chromosome end initiate synapsis independently of recombination (Dernburg et al., 1998). In contrast to nematodes, SC formation in yeast and mammals is dependent on the early steps of recombination (Giroux et al., 1989; Dernburg et al., 1998; Romanienko and Camerini-Otero, 2000), perhaps prolonging the phase of synapsis and/or delaying its onset. The extent of pairing at the time of synapsis may influence the timing of synapsis completion and could be very different among organisms. Organisms that are dependent on recombination for synapsis tend to use a subset of those recombination sites to initiate synapsis (Joyce and McKim, 2007; Tsubouchi et al., 2008; Pyatnitskaya et al., 2022). Therefore, completed chromosome synapsis depends on both the rate of elongation and the number of initiation sites used as well as the lengths of the chromosomes. Certain yeast strains backgrounds, including SK1, can reach full synapsis in less than 4 h (Padmore et al., 1991), whereas the BR strains spend approximately 11 h undergoing SC formation. Unless the rate of SC formation is significantly different in these two laboratory yeast strains, the shorter time spent synapsis suggests that other regulatory controls such as the number and timing of synapsis initiations, may be responsible.

### Threshold vs. meiotic progression models for synapsis initiation

We quantified Zip1-GFP accumulation in the nucleus to monitor cells during the active phase of SC assembly and disassembly. We found that chromosome synapsis initiated when Zip1-GFP levels reached comparable levels in both wild-type and *zip3Δ* cells, suggesting that a threshold of SC components accumulates before SC formation begins. It is also possible that rather than a threshold, reaching a particular stage of meiotic progression licenses SC formation and Zip1 levels are just coincidentally the same in wild type and mutant. Voelkel-Meiman et al. (2012) monitored synapsis in BR strains with 1–6 copies of Zip1 and found that as the copy number increased, synapsis started earlier. In a threshold model, higher levels of Zip1 in SK1 vs. BR strains might explain, in part, how SK1 starts synapsis earlier. It is also consistent with the fact that SK1 normally synapses in the presence of polycomplexes.

### Lower abundance of Zip1 in *zip3Δ* mutants

As seen in Figure 4B, in *zip3Δ* less overall Zip1 is seen compared to wild-type as time progresses. One possibility is that SC structures themselves stabilize/maintain abundance of Zip1—i.e., Zip1 that is incorporated into SC may be less likely to degrade than Zip1 floating in the nucleoplasm. Perhaps it is the SC structure that stabilizes Zip1 thereby promoting its accumulation. Another possibility is that a feedback loop exists such that more Zip1 is produced as more is incorporated into chromosomes.

### Final disassembly involves removal of SC from the ends

In contrast to the stochastic assembly of Zip1-GFP on individual chromosomes throughout the synapsis phase, the final SC disassembly happens all at once to all synapsed chromosomes. The concentration of Zip1 remains high until programmed SC disassembly, which is abrupt in wild type, and attenuated in *zip3Δ*. In both strains the SCs began rapid disassembly when Zip1 levels begin to decline (within 1.5 h for *zip3Δ*), suggesting that *zip3Δ* diploids retain programmed disassembly signals, but they may be compromised. Disassembly of the SC is monophasic with a similar rate to assembly. The loss of Zip1 occurs at the ends, the reverse of assembly. However, the possibility exists that in wild-type cells, disassembly may also occur interstitially, potentially at the sites of synapsis initiation. This is difficult to measure due to the apparent intensity changes that accompany a change in orientation of the SC.

### Abortive disassembly may be a way to correct synapsis

Unexpectedly, we encountered many SCs in *zip3Δ* nuclei that disassembled in advance of final disassembly when Zip1 protein is actively degraded. We refer to the disassembly of these SCs as “abortive disassembly” since these SCs fail to persist to the end of the synapsis phase. The abortive disassembly process represents SCs that are disassembling at the same time that others can be assembling, making it reminiscent of the “dynamic instability” phenomenon in microtubules (Kirschner and Mitchison, 1986). Most of these SCs were very short, but a few larger SCs were observed. Aborted SCs were also observed in wild-type meiosis, but examples were technically harder to identify because so many SCs are assembling at the same time, and it is likely a much rarer event. Abortive disassembly was not observed in the nematode (Rog and Dernburg, 2015). Our data reveal that ∼30% of the 5-h time courses (representing about a third of the synaptic period) had one or more abortive SCs, suggesting that in *zip3*Δ mutants, abortive SCs are fairly common. Consistent with our data, examination of fixed nuclei indicated that *zip3* meiotic nuclei did not gain as many SCs as wild type (Voelkel-Meiman et al., 2019). However, the dynamics of assembly and disassembly of SCs can only be revealed from live imaging, illuminating the wealth of data that can be uncovered from real-time imaging. One prediction for future *in vivo* studies is that abortive disassembly should be more frequent in hybrid strains for which there are a lot of polymorphisms.

We hypothesize that the aborted SCs are identified as defective or stalled SCs by a yet uncharacterized surveillance mechanism, and then targeted for disassembly. We speculate that many of the aborted SCs identified in *zip3*Δ mutants represent nascent SCs that were formed between non-homologous chromosomes. Zip3, with Fpr3, has been proposed to have a role in licensing SC formation at centromeres after recombination has initiated (Macqueen and Roeder, 2009). Consequently, when this license is defective as in *zip3 fpr3*, promiscuous SC formation occurs in *spo11* mutants, which are recombination-negative, and in haploids, which do not have homologs. *zip3 fpr3* double mutants attain wild-type levels of SCs but have low spore viability, implying that apparent full synapsis cannot rescue *zip3*. It seems likely that centromere-initiated synapsis, in the absence of regulation, is error-prone. This could explain why most of the aborted SCs were very short, since a lack of homology may slow down elongation or may be a signal for SC abortion or have some physical characteristic that lacks stability. Perhaps these short SCs, doomed to disappear, may not have established a robust central element. We can envision a scenario in which these short non-homologous SCs are tugged apart by the telomere-led movements during prophase. Perhaps by virtue of being non-homologous and relatively short, they are more vulnerable to telomeric pulls. Rather than invoking a sensing mechanism to seek out non-homology, perhaps the physical jerking of the chromosomes is enough to disrupt non-productive SCs. For those SCs that had attained significant length before they are aborted, they may be the result of entanglements with other chromosomes. Perhaps the pachytene checkpoint acts to prolong the synapsis phase in *zip3Δ* to allow time for entanglement resolution. In the future, potentially more elaborate FISH experiments like shown in Figure 1D will be able to test the hypothesis that the abortive disassembly events predominantly stem from non-homologous interactions.

During the past several decades, numerous genes involved in chromosome pairing and synapsis have been identified, and the protein architecture of the SC determined, yet many basic questions remain concerning the links between homology recognition, SC assembly, interlock resolution, recombination, and crossover distribution. The complex dynamics of meiosis are one reason that these questions have been difficult to answer. Genetic methods typically apply a constant-in-time perturbation and then probe the end-point result. The analysis of the dynamics of SC assembly and disassembly in real time provides a different view of the same events that have long been probed by genetic means, and has revealed unexpected features such as biphasic growth and abortive disassembly that had not been predicted on the basis of genetic analysis. Our work thus represents a step towards mechanistic understanding of meiosis as a dynamic process.

## Data Availability

The raw data supporting the conclusion of this article will be made available by the authors, without undue reservation.

## Supplementary material

SUPPLEMENTARY FIGURE S1–S3 SC movies in zip3*Δ* strains. 2D projection movies of the dynamic motion of *zip3Δ* SCs. 200 ms interval time series. S1-movie associated with Figure 2A. S2-movie with 9 SCs. S3-movie with 1 SC.

SUPPLEMENTARY FIGURE S4 Effects of nonoptimal imaging conditions. Preliminary experiments revealed that excessive exposure of the yeast cells to the 488 nm laser at a power greater than 30 µW results in phototoxic effects such that formation of a Zip1 aggregate occurs or that chromosomes adopt a fixed configuration and no longer appear subject to telomere-led pulls (top and middle panel). At our imaging conditions at 11 µW, bleaching rates were reduced and no aberrant chromosome behavior was observed (bottom panel). Frame numbers are indicated. Data was taken at 200 ms time intervals.

SUPPLEMENTARY TABLE S1 Yeast Strains.

SUPPLEMENTARY TABLE S2 SC lengths from monophasic and biphasic assembly.
