# Supplementary material for: Kinetic analysis of synaptonemal complex dynamics during meiosis of yeast Saccharomyces cerevisiae reveals biphasic growth and abortive disassembly
Source: Front Cell Dev Biol. 2023 Feb 6;11:1098468. doi: 10.3389/fcell.2023.1098468 (PMC9939684; doi:10.3389/fcell.2023.1098468)
Supplement: Supplementary file 2 [file Table1.DOCX]

**Table S1. Yeast strains**

| Strain Name | Ploidy | Relevant Genotype | Additional genotype |
| --- | --- | --- | --- |
| 1919-8B | a/α | *ZIP1/ZIP1 ZIP3/ZIP3* | *leu2-3,122 his4-260 ura3-1 ade2-1 thr1-4 lys2 trp1-289* |
| YMP104 | a/α | *ZIP1-GFP^700^@ZIP1/zip1::KAN, zip3::LYS2/zip3::LYS2* | *leu2-3,122 his4-260 ura3-1, thr1-4 lys2 trp1-289* |
| yCA660 | a/α | *Zip1-GFP(700)@ZIP1/∆zip1::KAN* | *leu2-3,122 his4-260 ura3-1 thr1-4 lys2 trp1-289* |
| JCF7001 | a/α | *ZIP1/∆zip1::KAN* | *leu2-3,122 his4-260 ura3-1 thr1-4 lys2 trp1-289* |
| JCF7002 | a/α | *zip3::LYS2/zip3::LYS2* | *leu2-3,122 his4-260 ura3-1 ade2-1 thr1-4 lys2 trp1-289* |
