# Supplementary figures and images for: Kinetic analysis of synaptonemal complex dynamics during meiosis of yeast Saccharomyces cerevisiae reveals biphasic growth and abortive disassembly
Source: Front Cell Dev Biol. 2023 Feb 6;11:1098468. doi: 10.3389/fcell.2023.1098468 (PMC9939684; doi:10.3389/fcell.2023.1098468)

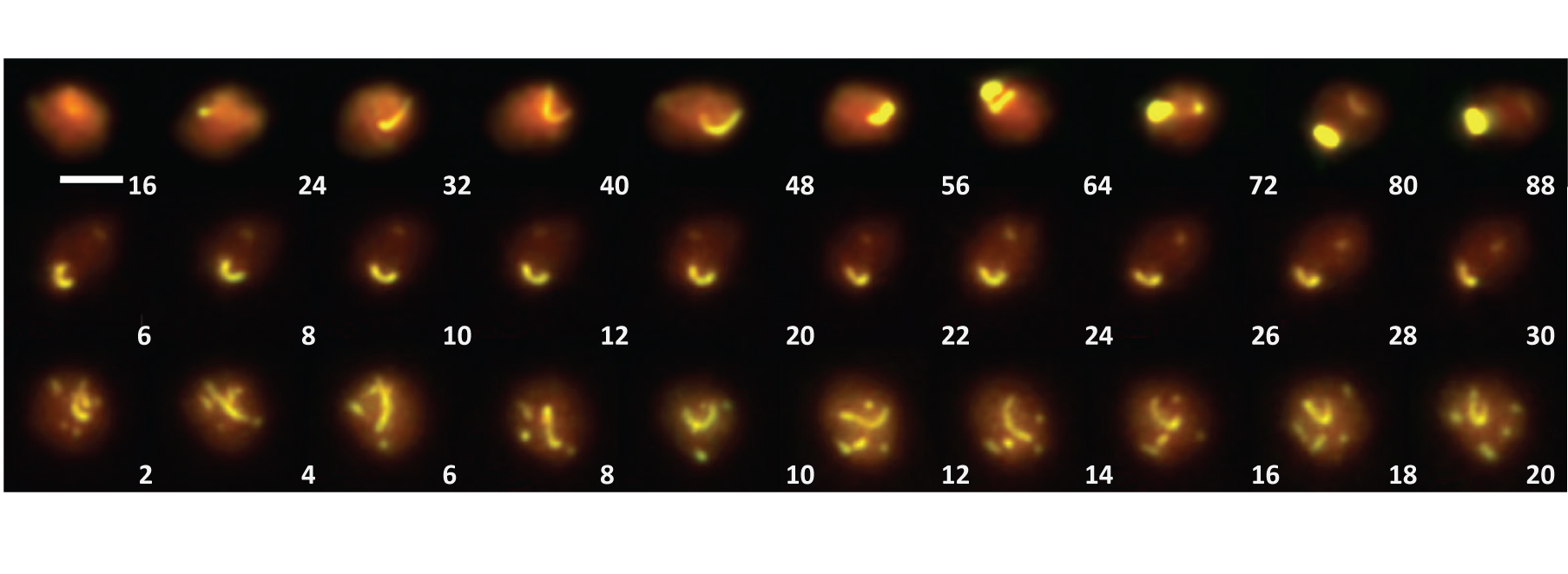

Supplement: Supplementary file 3 [file Image1.TIF]
