# Supplementary material for: Kinetic analysis of synaptonemal complex dynamics during meiosis of yeast Saccharomyces cerevisiae reveals biphasic growth and abortive disassembly
Source: Front Cell Dev Biol. 2023 Feb 6;11:1098468. doi: 10.3389/fcell.2023.1098468 (PMC9939684; doi:10.3389/fcell.2023.1098468)
Supplement: Supplementary file 4 [file Table2.DOCX]

**Table S2. SC lengths from monophasic and biphasic assembly**

| **SC length (µm)** | | | | |
| --- | --- | --- | --- | --- |
| Cell | Phase | First Growth Phase | Second Growth Phase | Final length |
| 35A29A | monophasic | 2.19 |  | 2.19 |
| 36B111C | monophasic | 1.79 |  | 1.79 |
| 36C25D_01 | monophasic | 2.06 |  | 2.06 |
| 36C30A | monophasic | 2.49 |  | 2.49 |
| 36H21D | biphasic | 1.25 | 0.90 | 2.15 |
| 36I11E | monophasic | 1.90 |  | 1.90 |
| 36I14B | monophasic | 1.42 |  | 1.42 |
| 36I53C | biphasic | 2.55 | 0.47 | 3.02 |
| 36I71B_01 | monophasic | 0.74 |  | 0.74 |
| 36I71B_02 | monophasic | 1.67 |  | 1.67 |
| 36I88A | monophasic | 1.95 |  | 1.95 |
| 36I95B | monophasic | 2.33 |  | 2.33 |
| 38A06B | monophasic | 1.28 |  | 1.28 |
| 38E50I | biphasic | 1.18 | 1.56 | 3.25 |
| 38G84A | monophasic | 2.44 |  | 2.44 |
| 38I79D | monophasic | 2.16 |  | 2.16 |
| 38I79E | biphasic | 1.25 | 1.38 | 2.63 |
| 38I93A | monophasic | 1.69 |  | 1.69 |
| 38I94B | monophasic | 2.57 |  | 2.57 |
| 38I120H | biphasic | 1.03 | 1.51 | 2.54 |
| 39C51F | monophasic | 2.60 |  | 2.60 |
| 39C056A | biphasic | 2.36 | 1.35 | 3.71 |
| 39C056C | biphasic | 1.09 | 0.80 | 1.89 |
| 39C068B | monophasic | 2.93 |  | 2.93 |
| 39C070A | monophasic | 2.15 |  | 2.15 |
| 39C091D | monophasic | 3.26 |  | 3.26 |
| 39C92E | monophasic | 1.39 |  | 1.39 |
| 39C106A | monophasic | 2.50 |  | 2.50 |
| 39C116B | monophasic | 2.49 |  | 2.49 |
| 39C180E | monophasic | 1.69 |  | 1.69 |
| 39C439A | monophasic | 1.68 |  | 1.68 |
| 39C461B | monophasic | 0.99 | 0.24 | 1.23 |
| 39C474E | monophasic | 1.62 |  | 1.62 |
